# 大麻中3种酚类成分测定专用固相萃取柱的设计与应用

**DOI:** 10.3724/SP.J.1123.2020.09025

**Published:** 2021-05-08

**Authors:** Shuchang SHEN, Shaohua LI, Li GUO, Weichao LÜ, Qiushi LI

**Affiliations:** 1.齐齐哈尔大学分析测试中心, 黑龙江 齐齐哈尔 161006; 1. Center of Analytical and Testing, Qiqihar University, Qiqihar 161006, China; 2.黑龙江省农业科学院大庆分院, 黑龙江 大庆 163316; 2. Daqing Branches of Heilongjiang Academy of Agricutural Sciences, Daqing 163316, China

**Keywords:** 超高效液相色谱, 大麻二酚, 大麻酚, Δ9-四氢大麻酚;, 大麻, 专用固相萃取柱, ultra-high performance liquid chromatography (UHPLC), cannabidiol, cannabinol, Δ9-tetrahydrocannabinol;, hemp, special solid phase extraction column

## Abstract

大麻中的主要成分大麻二酚(CBD)、大麻酚(CBN)和Δ9-四氢大麻酚(Δ9-THC)的含量决定了其性质和应用。在液相色谱分析中,由于大麻提取液中含有较多杂质,需要净化。该文基于大麻中CBD、CBN和Δ9-THC的结构特征及样品基质组成,根据中性氧化铝、硅酸镁和石墨化炭黑的不同表面特征,考察了这3种吸附剂对大麻提取液中叶绿素、多糖、高级脂肪酸酯及重金属离子的去除率和对3种大麻酚的回收率,确定了3种吸附剂的用量分别为1.80 g、0.15 g、0.05 g混合装填成的2 g/6 mL小柱为3种大麻酚类化合物测定的专用固相萃取柱。该小柱对大麻乙酸乙酯-甲醇提取液样品中CBD、CBN和Δ9-THC的回收率分别为98.9%, 95.7%和99.2%,对叶黄素、叶绿素a和叶绿素b的去除率分别为96.3%、99.2%和95.5%,对总糖的去除率为98.5%,对脂肪酸甘油酯的去除率为96.9%,对重金属离子的平均去除率为85.4%。优化了色谱分析条件,采用Eclipse Plus C18色谱柱(50 mm×2.1 mm, 1.8 μm),在1%乙酸水溶液-乙腈(30:70, v/v)流动相条件下等度洗脱,流速为0.5 mL/min,柱温为30 ℃,检测波长为210 nm,进样量为1 μL,在10 min内可完成样品分析。方法学考察表明,在0.5~50 mg/L范围内,CBD、CBN和Δ9-THC的液相色谱峰面积与其质量浓度呈良好的线性关系,相关系数(*R*^2^)分别为0.9983、0.9995和0.9981,检出限分别为0.45 μg/L、0.53 μg/L和0.38 μg/L,加标回收率为90.3%~97.0%、93.7%~95.6%、90.8%~96.1%,相对标准偏差(RSD)分别为2.2%~6.1%、4.1%~8.0%、2.4%~4.8%。研究结果表明,该文以中性氧化铝、硅酸镁和石墨化炭黑制作的复合型大麻酚类成分测定专用固相萃取柱在大麻植物中3种酚类化合物的测定中具有净化杂质、防止色谱柱污染的功能。由于大麻不同部位的化学成分存在差异,在后续的研究中,还要进一步考察小柱对其他杂质的去除情况,使得制备的固相萃取小柱更具有普适性。

## Abstract (en)

Cannabidiol (CBD), cannabinol (CBN), and Δ9-tetrahydrocannabinol (THC) are the most important components of hemp, whose concentrations determine the properties and applications of hemp. Hemp contains a large number of impurities, which must be removed from the extracting solution before determining the cannabinol contents by ultra-high performance liquid chromatography (UHPLC). Neutral alumina, magnesium silicate, and graphitized carbon black have different surface characteristics when used as adsorbents. The removal rates of pigments, total sugar, total fatty acid glyceride, and metal ions as well as the recoveries of the three cannabinols in the extraction solution were evaluated. The amounts of neutral alumina, magnesium silicate, and graphitized carbon black were 1.80 g, 0.15 g, and 0.05 g, respectively. The three adsorbents were mixed well and packed into a polypropylene pipe to prepare a special 2 g/6 mL solid phase extraction (SPE) column for determining the three cannabinol compounds in hemp. The chemical components of the hemp flowers and leaves were extracted with an ethyl acetate/methanol (9:1, v/v) mixture. After the extracting solution was allowed to pass through the SPE column, the recoveries of CBD, CBN, and Δ9-THC were 98.9%, 95.7%, and 99.2%, respectively. The removal rates of xanthophyll, chlorophyll a, and chlorophyll were 96.3%, 99.2%, and 95.5%, respectively. The removal rates of total sugar, total fatty glyceride, and metal ions were 98.5%, 96.9%, and 85.4%, respectively. In this study, the chromatographic conditions for analyzing the three cannabinol compounds were optimized. The cannabinol compounds were separated within 10 min on an Eclipse Plus C18 column (50 mm×2.1 mm, 1.8 μm) using a mobile phase consisting of 1% (v/v) acetic acid and acetonitrile (30:70, v/v) at a flow rate of 0.5 mL/min. The detection wavelength was set at 210 nm with a diode array detector, and the sample injection volume was 1 μL. Good linear relationships were observed between the mass peak areas and mass concentrations of CBD, CBN, and Δ9-THC in the range of 0.5-50 mg/L. The corresponding correlation coefficients (*R*^2^) were 0.9983, 0.9995, and 0.9981, while the detection limits were 0.45 μg/L, 0.53 μg/L, and 0.38 μg/L. The recoveries of CBD, CBN, and Δ9-THC were 90.3%-96.9%, 93.7%-95.6%, and 90.8%-96.1%, with relative standard deviations (RSDs) of 2.2%-6.1%, 4.1%-8.0%, and 2.4%-4.8%, respectively. The results were satisfactory, demonstrating that the special SPE column made of neutral alumina, magnesium silicate, and graphitized carbon black was well suited for the determination of the three cannabinol compounds in hemp.

大麻(*Cannabis sativa L.*)分为北亚种大麻(*Cannabis sativa ssp. sativa*)和南亚种大麻(*Cannabis sativa ssp. indica*);南亚种大麻具有精神活性,为禁止种植品种;北亚种大麻具有较高的经济利用价值,在造纸、纺织、功能保健食品、医药等行业用途广泛^[1,2,3]^。大麻酚类化合物是大麻植物中特有的最重要的成分,其中主要是大麻二酚(CBD)、大麻酚(CBN)和Δ9-四氢大麻酚(Δ9-THC)^[4,5]^。大麻二酚具有药理作用^[6,7]^, Δ9-四氢大麻酚是使人致幻成瘾的主要成分^[8]^,大麻酚是具有医疗用途的大麻素^[8]^。

国际上主要根据Δ9-四氢大麻酚和大麻二酚的含量及比值将大麻分为3种化学型:毒品型大麻、中间型大麻和工业大麻^[9]^。由于全球范围内已有多个国家将医用大麻全面合法化,促进了行业增长^[10,11,12]^。品种不同、生长环境不同的大麻中大麻酚类化合物的组成也不同,采取不同工艺条件获得的提取物中的大麻二酚、大麻酚和Δ9-四氢大麻酚的含量也不一致^[13,14,15]^。判定大麻及其提取物的性质及应用对监管部门来说非常重要^[16]^,这也给分析化学工作者提出了重要的研究课题。

大麻中CBD、CBN和Δ9-THC含量检测的最合适方法是液相色谱法。在目前报道的大麻中3种大麻酚测定的文献中,多数^[16,17,18,19]^是采用溶剂萃取、滤膜过滤,直接进行高效液相色谱测定,这些方法存在的主要缺点是没有对提取溶液进行很好的前处理。大麻化学成分复杂^[20,21,22,23]^,提取液中含有一定量的叶绿素、多糖、高级脂肪酸酯及重金属离子等成分。如果不对样品净化,大量的杂质峰会影响被测组分测定结果的准确性;一些难溶于流动相的有机物、金属盐以及某些过渡金属离子与流动相形成的配合物团簇离子^[24]^就会沉积在固定相中,杂质累积到一定程度后会形成新的伪固定相,从而改变色谱柱的性能,对色谱柱造成严重的损害^[25,26]^;有些杂质在紫外检测波长下不出现明显的吸收峰,但仍会出现色谱基线波动增加分析误差;为了防止有些杂质在柱中滞留,需延长分析时间。对大麻提取液进行净化是色谱分析工作者重要的研究课题。固相萃取(SPE)是一种将分析物与干扰组分有效分离的色谱分析样品前处理方法,是大麻化学成分分析中提取液净化的首选方法,但目前还没有用于大麻中CBD、CBN和Δ9-THC测定的专用固相萃取柱。商品化的固相萃取柱有多种类型,张旭等^[27]^选用中性氧化铝(alumina-N)固相萃取小柱对大麻的甲醇提取液进行了净化后液相色谱测定,并考察了Florisil、C8、C18、alumina-A(酸性氧化铝)、alumina-N、alumina-B(碱性氧化铝)不同填料的固相小柱对CBD、CBN、Δ9-THC的吸附率;李航麒等^[28]^对大麻的甲醇-正己烷提取液进行了alumina-N柱净化和HPLC测定。尽管以上文献均得出了经固相萃取净化处理后,提取液的颜色(深绿色)明显淡化的结论,但没有考察色素的去除率以及小柱对其他杂质的吸附性能。综合商品化固相萃取小柱填料的性能及有关文献的工作可见,单一吸附剂的固相萃取小柱无法解决上述大麻提取液中杂质的去除问题。

本文依据中性氧化铝、硅酸镁和石墨化炭黑的分子结构和表面特征,将3种吸附剂按最佳用量混合制成复合型专用固相萃取柱,呈现了极佳的吸附杂质的效果,对3种大麻酚的回收率高。净化后的样品经超高效液相色谱法(UHPLC)测定CBD、CBN、Δ9-THC的含量,结果准确可靠,重现性好,方法令人满意。

## 1 实验部分

### 1.1 主要仪器与试剂

1290 InfinityⅡ型超高效液相色谱仪、Eclipse Plus C18色谱柱(50 mm×2.1 mm, 1.8 μm,美国Agilent公司); Visiprep DL SPE型固相萃取装置(美国Supelco公司); DHG-9145A型电热鼓风干燥箱(上海一恒科学仪器有限公司); SK2200HP型超声波清洗仪(上海科导超声仪器有限公司); IKA RW20 Digital型电动搅拌机(德国IKA公司); Adventurer系列AR1140型电子分析天平(上海奥豪斯仪器有限公司); Lamda35紫外分光光度计、NEXION-350X电感耦合等离子体质谱联用仪(美国PE公司)。

乙腈(色谱纯,国药集团化学试剂有限公司);乙酸、乙酸乙酯(分析纯,天津市凯通化学试剂有限公司);甲醇(色谱纯,天津市光复精细化工研究所);大麻二酚标准溶液、大麻酚标准溶液、Δ9-四氢大麻酚标准溶液(1.0 mg/mL;美国Sigma-Aldrich公司);中性氧化铝(200~300目,国药集团化学试剂有限公司海拓实验室);硅酸镁(100~200目,国药集团化学试剂有限公司);石墨化炭黑(100~200目,上海熹恒生物科技有限公司)。

大麻花叶粉末(使用前过60目分样筛)由黑龙江省农业科学院大庆分院提供。

### 1.2 固相萃取小柱的制备

称取1.80 g中性氧化铝、0.15 g硅酸镁和0.05 g石墨化炭黑于圆底烧瓶中,加入5 mL乙醇,用电动搅拌机搅拌均匀,除去乙醇,将剩余物转移至瓷坩埚中,于180 ℃鼓风干燥箱中恒温干燥2 h。取6 mL聚丙烯管,将多孔聚乙烯筛板装入硬管底部,通过漏斗将填料加入垂直放置的聚丙烯管中,多次轻敲管壁使填料密实,将另一片多孔聚乙烯筛板装入聚丙烯管中并压实填料,填料上下表面平齐,制成2 g/6 mL固相萃取小柱。

### 1.3 标准溶液的配制

分别取大麻二酚、大麻酚和Δ9-四氢大麻酚标准溶液(1.0 mg/mL)各500 μL于10 mL容量瓶中,用甲醇定容至刻度,制成质量浓度均为50 mg/L的储备液。分别吸取标准储备液0.05、0.1、0.5、1.0、3.0 mL至5 mL容量瓶中,用甲醇定容,得到质量浓度为0.5、1.0、5.0、10、30、50 mg/L的大麻二酚、大麻酚和Δ9-四氢大麻酚系列标准溶液。

### 1.4 大麻酚类化合物的提取

取0.2 g大麻花叶粉末(精确至0.0001 g)于25 mL玻璃试管中,加入10 mL乙酸乙酯-甲醇混合溶剂(9∶1, v/v),超声提取30 min,静置10 min,待净化。

### 1.5 样品净化及分析

取5 mL乙酸乙酯-甲醇混合溶剂(9∶1, v/v)活化固相萃取小柱,在柱床湿润状态下,将1 mL提取液以0.5 mL/min速度过自制固相萃取小柱,再用1 mL乙酸乙酯-甲醇混合溶剂洗脱残留的被测物,流出液全部收集于玻璃试样瓶中,在40 ℃水浴温度下,用氮气将溶剂吹干,用1 mL甲醇溶解,经0.22 μm有机滤膜过滤后进行UHPLC分析。

### 1.6 色谱分析条件

色谱柱为Eclipse Plus C18柱(50 mm×2.1 mm, 1.8 μm,美国安捷伦公司),流动相为乙腈-1%乙酸水溶液(70∶30, v/v),等度洗脱,流速为0.5 mL/min,柱温为30 ℃,检测波长为210 nm,进样量为1 μL,运行时间10 min。

### 1.7 固相萃取填料的吸附性能实验

将中性氧化铝、硅酸镁和石墨化炭黑3种吸附剂按表1中的填料量制备6只复合型固相萃取小柱。分别移取一定量的大麻提取液,过0.22 μm有机相滤膜后,按1.5节操作步骤净化样品。

**表 1:** SPE柱中不同吸附剂的用量

Column No.	*m*(Neutral alumina)/g	*m*(Magnesium silicate)/g	*m*(Graphitized carbon black)/g
1	0.67	0.67	0.67
2	1.50	0.30	0.20
3	1.60	0.30	0.10
4	1.70	0.20	0.10
5	1.80	0.15	0.05
6	1.85	0.12	0.03

### 1.8 含量测定

色素 在446 nm(叶黄素)、649 nm(叶绿素b)和665 nm(叶绿素a)波长^[29]^下分别测定净化前和净化后溶液的吸光度,计算小柱对色素的去除率。

糖 分别移取一定量的大麻提取液和净化液,减压真空将溶剂挥发后,用水溶解剩余物并定容,采用硫酸-苯酚法^[30]^测定净化前和净化后溶液中总糖含量,计算小柱对糖类物质的去除率。

金属离子 通过ICP-MS^[31]^测定净化前和净化后溶液中Cr^3+^、Mn^4+^、Ni^2+^、Cu^2+^、Zn^2+^、Pb^2+^、As^3+^、Ag^+^、Cd^2+^的含量,计算小柱对样品中金属离子的去除率。

脂肪酸甘油酯 将净化前和净化后溶液通过减压真空去除溶剂后,经氢氧化钾-甲醇溶液皂化、盐酸酸化、提取、甲酯化后,利用气相色谱法测定脂肪酸甲酯^[32]^的含量,计算小柱对样品中脂肪酸甘油酯的去除率。

## 2 结果与讨论

### 2.1 色谱条件的选择及样品净化的意义

由于检测器设定的波长为210 nm,乙腈截止使用波长(190 nm)比甲醇的(205 nm)小,因此,乙腈作流动相对被测组分定量干扰小。考察不同比例乙腈-1%乙酸水溶液(10∶90、30∶70、50∶50、70∶30, v/v)作为流动相时的色谱分离效果,结果表明乙腈-1%乙酸水溶液的体积比为70∶30时,峰形对称。

在1.6节色谱条件下,首先对未经净化的样品进行分析,操作过程中发现,随着进样次数的增加,色谱柱压渐渐升高,基线波动较大,最后出现了色谱峰拖尾、变宽、分叉的现象。用甲醇、乙腈和水/甲醇分别冲洗色谱柱后,基线波动减小,但柱压降低幅度很小,色谱峰峰形基本未变。采用新的色谱柱,将样品净化然后进行分析,在与未净化样品的进样量及分析次数相同的情况下,未出现上述现象,色谱图见图1。实验结果说明:通过增加流动相洗脱时间可以解决有些有机物杂质在柱中滞留时间长的问题;但有些杂质与流动相形成沉淀,残留在色谱柱头堵塞筛板,导致色谱柱无法再生。因此,对大麻提取液样品进行净化十分必要。

**图 1:**
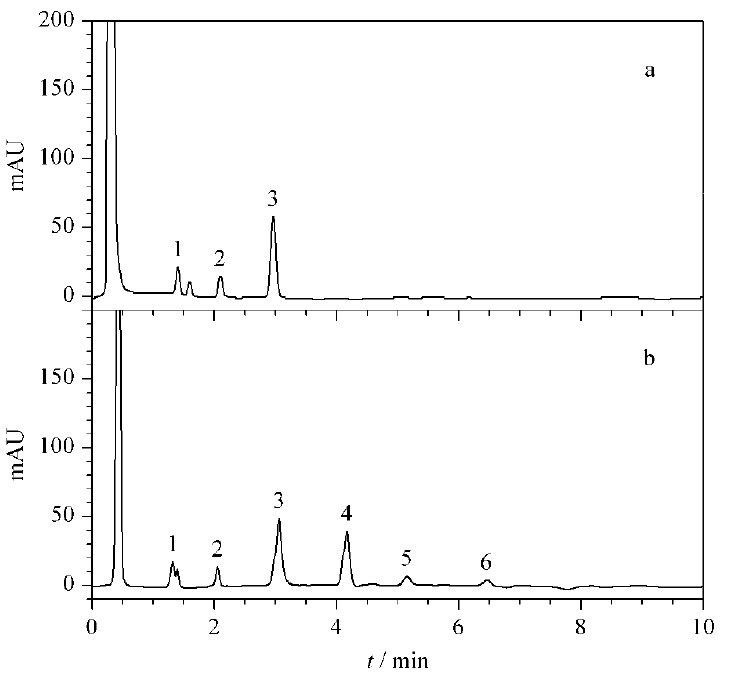
(a)净化样品和(b)未净化样品进样100次时的色谱图

### 2.2 萃取溶剂的选择

大麻二酚、大麻酚和Δ9-四氢大麻酚溶于甲醇、乙酸乙酯、氯仿、乙醚等多种有机溶剂中。甲醇为极性有机溶剂,相对分子质量小、易挥发,与植物纤维亲和力强,易于渗透到细胞结构中,萃取能力强。乙酸乙酯为弱极性有机溶剂,易挥发,毒性低,对有机物溶解能力强,但在植物纤维中的渗透性比甲醇弱。以甲醇单一溶剂提取大麻酚类化合物时,不利于吸附剂对提取液中极性杂质的保留,因此采用乙酸乙酯-甲醇混合溶剂(9∶1, v/v)作为萃取剂,萃取效率高,固相萃取净化效果好。

### 2.3 固相萃取填料的吸附性能

在使用固相萃取法对大麻的提取液样品净化时,可以选择强极性的填料去除其中的极性分子,也可以选择非极性的填料去除其中的非极性分子,还可以选择具有离子交换功能的填料去除金属离子及物理吸附作用去除某些物质。

对于中性氧化铝、硅酸镁和石墨化炭黑复合固相萃取小柱,3种吸附剂的用量和小柱的体积要依据对样品的净化效果、被测组分的回收率和被测组分的含量而确定。如果其中2种填料的用量不变,只改变1种填料用量,将会出现以下结果:中性氧化铝的用量增加,会大幅度提高小柱对糖类物质和脂肪酸甘油酯的去除率,而其他杂质的去除率也会有所增加,但不会降低小柱对3种大麻酚类化合物的回收率;硅酸镁用量的改变对重金属离子的去除率影响最大,其他杂质的去除率也有一定的变化,同时对3种大麻酚类化合物的回收率也有一定的影响。石墨化炭黑用量增加,色素的去除率迅速增加,同时直链脂肪酸酯的去除率较快增加,3种大麻酚类化合物的回收率也有较大的降低。

本文先设定总填料量为2 g,将3种吸附剂按等量配比(各0.67 g)制成固相萃取小柱,净化实验结果表明:3种大麻酚类化合物的回收率只有30%左右,叶黄素、叶绿素a和叶绿素b的去除率高于97%,总糖的去除率只有51%,总脂肪酸甘油酯的去除率为66%,重金属离子的吸附率为90%左右。因此,本文设计了表1中的6种填料配比,制备复合型固相萃取小柱,考察各小柱的吸附性能。固相萃取小柱对3种大麻酚类及主要杂质的去除率见图2。

**图 2:**
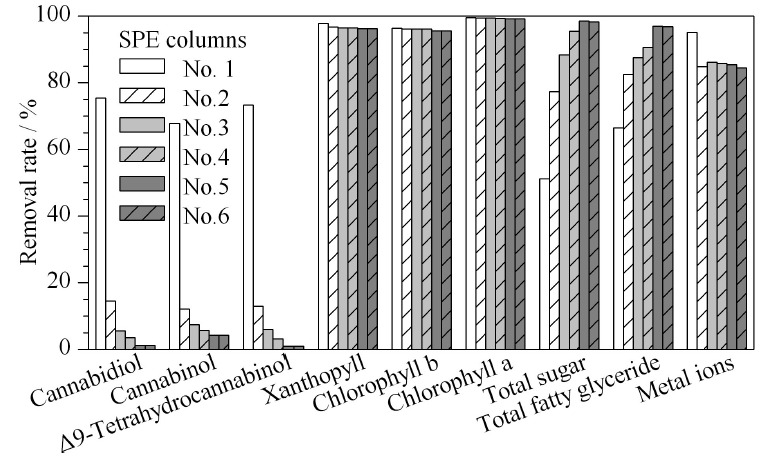
6种不同配比复合填料固相萃取柱对3种大麻酚类化合 物、色素、总糖、总脂肪酸酯及金属离子的去除率

实验发现,分层填装的小柱对提取液中组分去除率的重现性比3种填料均匀混合差。原因可能是石墨化炭黑用量较少,分层填装时出现了柱内不同位置的高度不一致所致。

由实验数据可知,中性氧化铝1.80 g、硅酸镁0.15 g、石墨化炭黑0.05 g制成的2 g/6 mL固相萃取小柱性能最佳,确定为大麻中3种酚类成分测定专用固相萃取柱。该小柱对大麻乙酸乙酯-甲醇提取液样品中CBD、CBN和Δ9-THC的回收率分别为98.9%、95.7%、99.2%,对叶黄素、叶绿素a和叶绿素b的去除率分别为96.3%、99.2%和95.5%,对总糖的去除率为98.5%,对脂肪酸甘油酯的去除率为96.9%,对重金属离子的平均去除率为85.4%。

### 2.4 商品固相萃取小柱的应用效果

分别选用迪科马科技有限公司制造的用于调味品中苏丹红分析的商品ProElut SDH复合型专用固相萃取柱(1 g/6 mL)及天津艾杰尔-飞诺美公司制造的用于茶叶和中草药农残分析的商品Cleanert TPT和Cleanert TPH复合型专用固相萃取柱(1 g/6 mL),对大麻提取液进行净化,实验数据见表2。

**表 2:** 商品固相萃取小柱对3种大麻酚类化合物、色素、总糖、总脂肪酸酯及金属离子的去除率

Column	Removal rates/%				
	Cannabinols	Pigments	Total sugar	Total fatty glyceride	Metal ions
ProElut SDH	63.32	26.82	18.92	89.13	7.234
Cleanert TPT	78.17	96.23	90.35	36.13	42.36
Cleanert TPH	79.03	97.03	90.68	35.83	43.53

实验数据可见,3种小柱对大麻提取液的净化效果均不够理想,同时对3种大麻酚也具有很强的吸附作用,不能准确定量。

### 2.5 标准曲线与样品检测结果

按照1.6节进行实验,以目标物的峰面积(*y*)对质量浓度(*x*)进行线性回归分析,在0.5~50 mg/L的线性范围内,测得标准溶液CBD、CBN、Δ9-THC的线性良好。

由色谱峰面积得到大麻提取液中CBD、CBN和Δ9-THC的浓度,并计算大麻中3种大麻酚的含量。按3倍噪声对应的样品浓度测定检出限。实验结果见表3。

**表 3:** CBD、CBN和Δ9-THC的线性回归方程和检出限

Component	Linear range/ (mg/L)	Linear equation	*R* ^2^	LOD/ (μg/L)
CBD	0.5-50	*y*=8.998*x*-3.899	0.9983	0.45
CBN	0.5-50	*y*=9.914*x*-4.813	0.9995	0.53
Δ9-THC	0.5-50	*y*=13.179*x*-1.505	0.9981	0.38

*y*: peak area; *x*: mass concentration, mg/L.

取5份0.1 mL大麻提取液(每份中CBD、CBN和Δ9-THC的质量均为1.624、0.3900和5.144 μg)于5个试剂瓶中,分别加入0.02、0.05、0.1、0.2、0.4 mL标准溶液(CBD、CBN和Δ9-THC含量均为100 mg/L);另取4份0.5 mL大麻提取液(每份中CBD、CBN和Δ9-THC的质量均为8.125、1.950和25.72 μg)于4个试剂瓶中,分别加入0.02、0.05、0.1、0.2 mL标准溶液(CBD、CBN和Δ9-THC含量均为100 mg/L),用提取溶剂定容至1 mL。按1.5节步骤操作,平行测定5次。根据实验数据计算加标回收率,实验结果见表4。

**表 4:** CBD、CBN、Δ9-THC的加标回收率和相对标准偏差(*n*=5)

Component	Background/ μg	Added/ μg	Found/ μg	Recovery/ %	RSD/ %
CBD	1.624	2	3.466	90.3	5.3
		5	6.484	91.4	4.9
		10	11.56	95.8	3.9
		20	21.57	96.5	6.1
		40	41.51	92.7	5.5
	8.125	2	9.349	92.3	2.2
		5	12.17	92.7	3.0
		10	17.58	97.0	2.7
		20	25.57	90.9	2.9
CBN	0.3900	2	2.365	93.7	8.0
		5	5.366	93.9	4.6
		10	10.37	95.6	7.8
		20	20.37	94.5	7.5
		40	40.37	94.3	7.4
	1.950	2	3.732	94.5	4.5
		5	6.552	94.3	5.3
		10	11.29	94.4	5.6
		20	20.64	94.0	4.1
Δ9-THC	5.144	2	6.671	90.8	3.4
		5	9.933	95.9	3.1
		10	14.71	91.6	4.8
		20	24.93	96.1	3.3
		40	44.82	93.8	3.4
	25.72	2	25.41	91.6	2.5
		5	28.51	92.8	3.1
		10	33.38	93.4	2.7
		20	43.58	95.3	2.4

## 3 结论

中性氧化铝、硅酸镁和石墨化炭黑具有不同的表面特征,以1.80 g+0.15 g+0.05 g混合后装填成2 g/6 mL专用固相萃取小柱,对乙酸乙酯-甲醇溶剂提取大麻花叶的混合液中的叶绿素、多糖、重金属离子和脂肪酸酯具有优良的吸附性能,实现了对色谱分析样品的净化,通过UHPLC测定大麻二酚、大麻酚和Δ9-四氢大麻酚含量,分析时间短,测定结果准确、灵敏度高。
